# Testicular self-examination and testicular cancer: a cost-utility analysis

**DOI:** 10.1002/cam4.318

**Published:** 2014-08-08

**Authors:** Michael Aberger, Bradley Wilson, Jeffrey M Holzbeierlein, Tomas L Griebling, Ajay K Nangia

**Affiliations:** 1 Department of Urology, University of Kansas Medical CenterKansas City, Kansas, 66160; 2 The Landon Center on Aging, University of Kansas Medical CenterKansas City, Kansas, 66160

**Keywords:** Cost analysis, prevention, screening, self-examination, testis cancer

## Abstract

The United States Preventive Services Task Force (USPSTF) has recommended against testicular self-examinations (TSE) or clinical examination for testicular cancer screening. However, in this recommendation there was no consideration of the significant fiscal cost of treating advanced disease versus evaluation of benign disease. In this study, a cost-utility validation for TSE was performed. The cost of treatment for an advanced-stage testicular tumor (both seminomatous and nonseminomatous) was compared to the cost of six other scenarios involving the clinical assessment of a testicular mass felt during self-examination (four benign and two early-stage malignant). Medicare reimbursements were used as an estimate for a national cost standard. The total treatment cost for an advanced-stage seminoma ($48,877) or nonseminoma ($51,592) equaled the cost of 313–330 benign office visits ($156); 180–190 office visits with scrotal ultrasound ($272); 79–83 office visits with serial scrotal ultrasounds and labs ($621); 6–7 office visits resulting in radical inguinal orchiectomy for benign pathology ($7,686) or 2–3 office visits resulting in treatment and surveillance of an early-stage testicular cancer ($17,283: seminoma, $26,190: nonseminoma). A large number of clinical evaluations based on the TSE for benign disease can be made compared to the cost of one missed advanced-stage tumor. An average of 2.4 to 1 cost benefit ratio was demonstrated for early detected testicular cancer versus advanced-stage disease.

## Introduction

Testicular cancer remains the most common solid malignancy in men between 15 and 34 years of age [1]. The age-adjusted incidence rate in the United States is 5.5 cases per 100,000 men per year with ∼8000 new cases and 370 deaths in 2013 [1]. The lifetime risk of testicular cancer is 0.39% or 1 in 260 [1]. The 5-year median relative survival by stage at diagnosis is 99% for cancer confined to the testis but drops to 74% for metastatic disease [1].

Two-thirds of nonseminomatous germ cell tumors (NSGCTs) and 15% of pure seminomas present with regional or distant metastases. Symptoms associated with metastatic disease occur as the presenting complaint in 10% to 20% of cases [2]. Reasons for late presentation include lack of early symptoms; lack of education about the significance of testicular masses; reluctance to seek evaluation of palpable and/or painful testicular masses; poor access to care; and lack of accuracy of testicular examination alone, with low sensitivity and specificity even if performed by a clinician [3–5]. Despite these facts, there is no formal screening algorithm for testicular cancer.

In 2004, the USPSTF made a Grade D recommendation for testicular self-examination (TSE) or clinical evaluation to screen for testicular cancer in asymptomatic males and then reaffirmed this recommendation in 2009 and again in 2011 [6, 7]. Grade D is defined as “moderate or high certainty that the service has no net benefit or that the harms outweigh the benefits”. The USPSTF recommendation was based on the insufficient evidence to demonstrate a screening advantage of TSE for early detection of disease; concern about over investigation and treatment of false-positive examinations; and the low incidence and high success rates of various treatments for testicular cancer. However, none of the articles reviewed by the panel met inclusion criteria to study TSE as a screening tool [6–10]. There was also no cost validation for the USPSTF recommendation against TSE in spite of national estimates placing a $21.8 million dollar price tag on the total cost of treating testicular cancer in 2000 [11, 12]. A cost analysis remains an essential component to the principles of screening [13, 14]. The objective of our study was to determine the potential fiscal ramifications of TSE for various real world testicular cancer scenarios using a national cost standardization model.

## Methods

**Figure 1:**
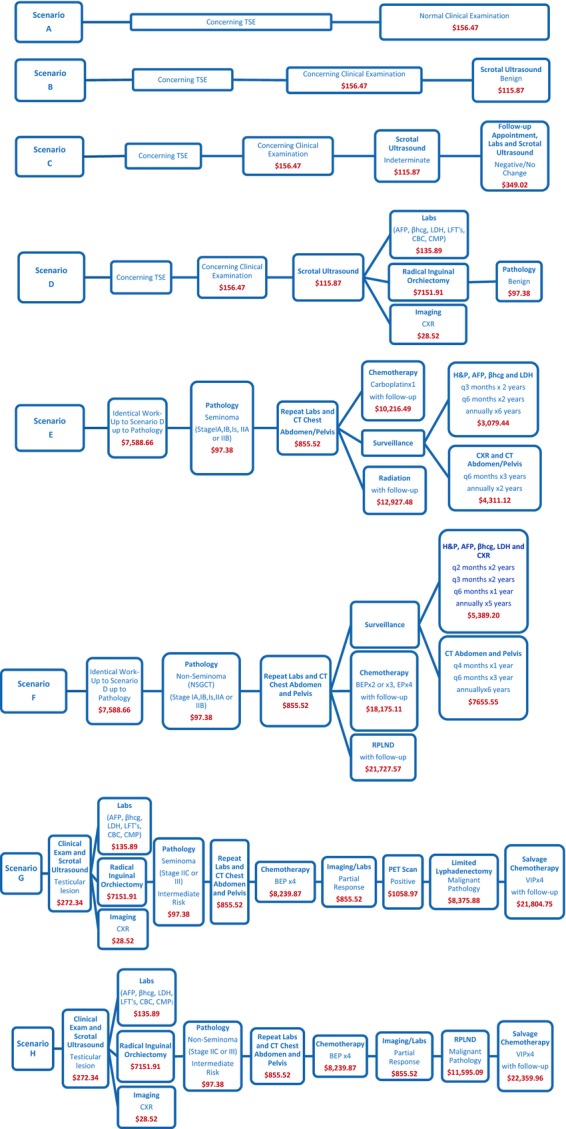
Scenarios; benign (A, B, C, D) and malignant (E, F, G, H).

We created eight common clinical scenarios involving the evaluation and treatment of a testicular mass identified during self-examination: four benign (scenarios A–D); two localized or regional malignant (scenarios E and F); and two advanced-stage testicular cancer (scenarios G and H) (Fig. 1). We focused on the clinical assessment of seminomatous and NSGCTs as these comprise around 98% of all primary testicular neoplasms. Cancer staging was performed according to the American Joint Committee on Cancer TNM system. Surveillance and treatment protocols from the 2013 National Comprehensive Cancer Network (NCCN) guidelines for testicular cancer were reviewed and applied for the modeling and analysis.

Cost analysis was based on the Medicare reimbursements. Most of the costs were determined using Current Procedural Terminology (CPT) codes and the par amount from the 2013 Kansas Medicare Physician Fee Database. Laboratory costs were obtained using CPT codes from the 2013 Medicare Clinical Lab Fee Schedule for Eastern Kansas (Locality 15). Chemotherapy drug costs were obtained using the January 2013 Payment Allowance Limits for Medicare Part B Drugs for the appropriate Healthcare Common Procedure Coding System (HCPCS) codes. In the various scenarios involving a testicular malignancy, we estimated the average cost of surveillance for 10 years following initial diagnosis and treatment.

The total cost of a stage IA or IB seminoma was broken down into surveillance, chemotherapy and radiation-specific groups, whereas the total cost of a stage IA or IB nonseminoma was sub-divided into surveillance, chemotherapy, and retroperitoneal lymph node dissection (RPLND)-specific groups. The cost of a stage IS, IIA, or IIB seminoma was included in the radiation-specific group cost. The cost of Stage IS, IIA, or IIB nonseminoma was divided into either chemotherapy or RPLND-specific groups. The average cost of the surveillance, chemotherapy, and radiation treatment arms (including stage IA, IB, IS, IIA, and IIB seminomas) was reported for scenario E. The surveillance protocol used for scenario E consisted of a clinical examination with tumor markers every 3 months for the first 2 years, every 6 months for the following 2 years and then annually for the next 6 years. Imaging consisted of a CT scan of abdomen/pelvis with chest x-ray every 6 months for the first 3 years and then annually for the following 2 years.

The average cost of the surveillance, chemotherapy, and RPLND treatment arms (including stage IA, IB, IS, IIA, and IIB nonseminomas) was reported for scenario F and the length of hospital stay accounted for following RPLND was 3 days (conservative estimate). The surveillance protocol used for scenario F consisted of a clinical examination with tumor markers and a chest x-ray every 2 months for the first 2 years, every 3 months for the following 2 years, every 6 months for the following year, and then annually for the following 5 years. Additional imaging consisted of a CT scan of the abdomen/pelvis every 4 months for the first year, every 6 months for the following 3 years, and then annually for the following 6 years.

The total cost for an advanced-stage seminoma (stage IIC or III) was calculated assuming initial intermediate risk, partial response to chemotherapy with residual masses, a limited resection of these masses and then salvage chemotherapy (scenario G). The surveillance protocol used for scenario G consisted of clinical examination, tumor markers, and chest x-ray every 2 months for the first year, every 3 months for the following year, every 6 months for the following 2 years, and then annually for the following 6 years. Additional imaging consisted of CT abdomen/pelvis every 6 months for the first 2 years and then annually for the following 8 years. The total cost for an advanced-stage nonseminoma (Stage IIC or III) was calculated assuming initial intermediate risk, partial response to chemotherapy with residual masses, RPLND, and then salvage chemotherapy (scenario H). The surveillance protocol used for scenario H consisted of clinical examination, tumor markers, and chest x-ray every 2 months for first 2 years, every 3 months for the following 2 years, every 6 months for the following year, and then annually for the following 5 years. Additional imaging consisted of CT abdomen/pelvis every 4 months for the first year, every 6 months for the following 3 years, and then annually for the following 6 years.

## Results

**Figure 2:**
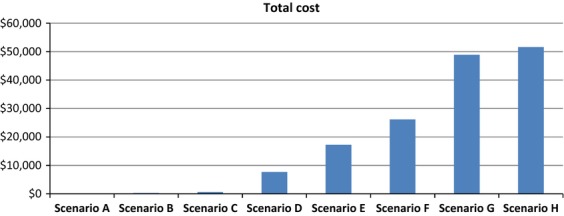
Total cost of each scenario.

Breakdown of costs for each step of treatment with scenarios A through H are shown in Figure 1. A more detailed breakdown is shown in the Table S1. The total treatment/surveillance cost for an advanced-stage seminoma and NSGCT is $48,877 and $51,592, respectively (scenarios G and H). The cost of one level 4 new patient office visit presenting to one provider, for example, primary care physician, urologist, or oncologist with a normal clinical examination prompted by concern on TSE is $156 (scenario A). An office visit with an abnormal clinical examination with resulting normal scrotal ultrasound is $272 (scenario B). Subsequent serial scrotal ultrasounds with tumor marker laboratory testing increased the cost to $621 (scenario C) and eventual radical inguinal orchiectomy for benign pathology costs $7,686 (scenario D). The average cost of the detection, treatment, and surveillance for an early-stage seminoma and NSGCT is $17,283, and $26,190, respectively, (scenarios E and F) with patients electing to undergo postorchiectomy chemotherapy or RPLND further increasing the cost (Fig. 2).

Based on the type of testicular cancer (NSGCT vs. seminoma), the total treatment cost for an advanced-stage testicular cancer equaled the cost of 313–330 (average 322) benign office visits (scenario A); 180–190 (average 185) office visits with scrotal ultrasound (scenario B); 79–83 (average 81) office visits with tumor marker laboratory testing and serial scrotal ultrasounds (scenario C); or 6–7 office visits resulting in radical inguinal orchiectomy for benign disease (scenario D). In terms of malignant disease; the detection, treatment, and surveillance of 2–3 (average 2.4) early-stage testicular cancers (scenarios E and F) equaled the cost of one advanced disease treatment with subsequent surveillance (scenarios G and H). In comparison, 125–168 (average 147) TSEs with negative clinical evaluations can be performed for the cost of treatment of one localized or regional testicular cancer.

Data from SEER indicate that of the 8000 new testicular cancers diagnosed in 2013, ∼12% presented with advanced/distant metastatic disease. This equates to 960 cases per year, the cost of which will follow scenarios G and H as outlined. This suggests that an average of 309,120 negative clinical examinations (322 × 960) following scenario A could be performed in 1 year for the cost of all the distant metastatic disease treated in 2013. If the remaining 7040 cancers are defined as localized or regional, then they will follow the treatment and cost algorithms according to scenarios E or F. This would equate to 1.03 million (7040 × 147) negative clinical evaluations for all the localized/regional cancers treated in 2013. This indicates that a total of 1.344 million false-positive TSEs with a negative clinical examination could be performed for the cost of all new testicular cancer cases diagnosed and subsequently treated in 2013.

In terms of cost alone, 960 advanced metastatic cases equated to approximately $48 million for the treatment of testicular cancer in 2013, when we calculated $50,000 spent per case if averaged between seminoma and NSGCT for scenarios G and H. For the remaining 7040 cancers in 2013, the cost could be estimated according to scenarios E and F with an average cost between seminoma and NSGCT of $21,737 per case. This equates to a cost for localized and/or regional treatment of $153 million in 2013. The total cost of testicular cancer treatment in 2013, by Medicare calculation alone, is estimated at $201 million.

## Discussion

Testicular cancer remains the most common solid malignancy in young males. Delays in diagnosis can dramatically impact survival with 5-year median relative survivals dropping from 99% for cancer confined to the testis to 74% for metastatic disease [1]. Moul et al. demonstrated a decrease in survival for germ cell tumors with greater than 16 weeks of delay in diagnosis [15]. Diagnostic delays are a well-recognized source of morbidity with a heavier burden of treatment and increased risk of later cardiovascular disease, pulmonary toxicity, and infertility.

With a lack of evidence examining the issue of screening for testicular cancer, the objective of our study was to determine the potential fiscal ramifications of TSE performed correctly, incorrectly or not at all and presenting with localized and advanced testicular cancer. We created practical clinical scenarios that are commonly encountered by any provider performing a scrotal exam. Since most men have testicular cancer only once in their life time it seems appropriate to compare fiscal effectiveness for a clinical evaluation of an abnormal TSE to the lifetime risk, which is 1 in 260 men. Based on the 313–330 false-positive or negative clinical evaluation visits for one advanced seminoma and NSGCT, respectively, it suggests that screening might be cost effective relative to a presentation of cancer as advanced disease. This was not seen with the ratio of 125 and 168 negative office visits to the cost of treatment of one early/localized and potentially early detected cancer.

The study accounted for the possibility of a clinician being defensive or unsure/inexperienced with a testicular physical exam finding as presented in scenario B. Bosl et al. showed that the longest period of diagnostic delay was actually due to the physicians in two-thirds of cases [5]. Scrotal ultrasound improves the error rate for the detection of a cancer compared to TSE or clinical examination alone with a sensitivity and specificity of 98% and 66.7%, respectively [16]. We showed that 180–190 visits with scrotal ultrasound cost the same as detection, treatment, and follow-up for one advanced testis cancer. The lack of specificity with ultrasound was considered in scenarios C and D. We included the possibility of an indeterminate finding on serial ultrasounds and/or subsequent exploratory surgery for an outcome that was benign and, therefore, a false positive finding for testis cancer. Despite the false positive and invasive nature of scenarios C and D, there were 79–83 evaluations and 6–7 surgeries for the cost of one late presentation of testicular cancer. These two scenarios are examples of overevaluation and treatment with associated time and psychological consequences. Palpable testicular and intratesticular masses that remain indeterminate will always continue to require monitoring and occasionally surgical exploration. We did not account for the scenario where inguinal exploration with intraoperative ultrasound to identify an intratesticular lesion and subsequent frozen section was benign and testicle sparing, since we wanted to consider the most common scenarios that present, especially in males with two testicles. Management of men with a solitary testis and testis sparing was beyond the scope of our modeling. Also, there is no database currently that would allow us to determine the number of men that fall into these groups on a national level. The best we can do is to extrapolate from limited studies. Carmignani et al. showed that 98% of ultrasounds for intratesticular lesions were benign/false positive [10]. It is important to remember that TSE is free and some false-positive findings have a possible significance, for example, varicocele with testicular atrophy, infertility, and/or pain.

We showed that treatment of one early or regional testicular cancer is half the cost of an advanced case. We also estimated that the total cost of testicular cancer treatment in 2013 was $201 million. Of this, approximately $48 million would be for treatment of advanced metastatic disease and $153 million for localized and/or regional treatment. This is a significant difference or increase from the estimate published in 2000 [13, 14]. The 12% incidence of advanced cancer in 2013 accounted for 24% of the overall cost of all testicular cancer care.

There were limitations of our study. We opted to use Medicare since it is the only national cost standard in the USA. We were aware that most men in the highest incidence age group will not be covered under Medicare, but this was the most practical method to standardize reimbursement. Reimbursement does not correctly represent true cost but we did not have access to this confidential data from our institution which varies between regions based on the contracts with vendors and insurance companies. Due to inconsistencies in hospital master charge lists and real costs, reimbursement by Medicare was considered the best standard. The Healthcare Cost and Utilization Project (HCUP) does not cover the scenarios presented. We worked with our regional Medicare payment scheme but are aware of regional variations and possible higher reimbursement according to the Geographic Practice Cost Index (GPCI). We did not average the cost across the USA, since the difference was not felt to be significant enough to warrant this extra analysis. Medicare reimbursements are often 20–40% below private insurances and as such we may have undervalued our cost analysis. We also did not account for future inflation in costs. Medicare reimbursement is prone to the least degree of variation overtime. We only factored in one new patient visit based on presentation to one provider, whether oncologist, urologist, or primary care provider. Adding additional one or two consults would not have changed overall cost or ratios significantly. We also did not calculate the cost of all possible scenarios with associated subtleties of cost. We only considered the most common presentations. We were unable to factor into our analysis the costs associated with lost work and lost or reduced fertility resulting from any treatment especially for advanced disease, with extensive chemotherapy and inpatient postoperative care after salvage RPLND. Furthermore, given the young age of the population, an analysis of lost quality-adjusted life years would demonstrate a greater cost. Anxiety from over investigation is mentioned as one of the USPSTF's concerns with TSE as a screening tool. We did not factor in the cost of anxiety induced from either management of a false-positive TSE or diagnosis of localized/metastatic cancer. This is a relatively intangible cost, unless related to time off work or mental health visits.

Another limitation of the model was that we had to make some assumptions: men actually performed a TSE; they found an abnormality correctly or incorrectly with or without early or late symptoms; and they sought medical evaluation. We had to make an assumption that men will present with an abnormal TSE or constitutional symptoms from metastatic disease and get detected for the cancer, early or late. Although these are assumptions, they are in fact exactly how patients present anyway. We could not directly account for intratesticular masses presenting with metastatic disease, but the regional or advanced diseases scenarios pick up this possibility since this is how they will eventually present.

Although cost alone does not equate to screening it does serve as a means to determine how many men can be seen. Testicular cancer is not preventable and may be very treatable as indicated by the USPSTF, but has significant morbidity and mortality despite this if detected late. Care of advanced disease is always going to cost more medically, psychologically, and financially than localized disease and it is important to reduce this cost, morbidity, and mortality through early detection and treatment. How that is achieved is difficult to determine without patient awareness and continued evidence-based research to validate early evaluation and detection, even if screening is not proven yet.

## Supporting Information

**Table S1.** Detailed breakdown of costs
