# Supplementary material for: Testicular self-examination and testicular cancer: a cost-utility analysis
Source: Cancer Med. 2014 Aug 8;3(6):1629–34. doi: 10.1002/cam4.318 (PMC4298389; doi:10.1002/cam4.318)
Supplement: Supplementary file 1 — Table S1. Detailed breakdown of costs [file cam40003-1629-sd1.pdf]

Appendix: Detailed breakdown of costs.

Scenario A	Finding	Medicare Costs ($)
TSE-felt something		
Level IV new patient	nothing on clinic exam	156.47
PRN		
**Total**		**156.47**
**Scenario B**	**Finding**	**Medicare Costs ($)**
TSE-felt something		
Level IV new patient	concerning clinical exam	156.47
Scrotal US	benign	115.87
PRN		
**Total**		**272.34**
**Scenario C**	**Finding**	**Medicare Costs ($)**
TSE-felt something		
Level IV new patient	concerning clinical exam	156.47
Scrotal US	questionable	115.87
AFP	normal	23.06
bHCG	normal	70.98
LDH	normal	8.30
LFT's	normal	11.23
CBC	normal	10.69
BMP	normal	11.63
CXR	normal	28.52
Follow-up Scrotal US	unchanged	115.87
Level III return		68.74
PRN		
**Total**		**621.36**
**Scenario D**	**Finding**	**Medicare Costs ($)**
TSE-felt something		
Level IV new patient		156.47
Scrotal US	intra-testicular lesion	115.87
AFP	normal	23.06
bHCG	normal	70.98
LDH	normal	8.30
LFT's	normal	11.23
CBC	normal	10.69
BMP	normal	11.63
CXR	normal	28.52
Rad Ing Orch		479.19
Anesthesia-General		363.72
Peri-operative services		6309.00
Pathology	benign	97.38
**Total**		**7686.04**

Scenario E	Finding	Medicare Costs ($)
TSE-felt something		
Level IV new patient		156.47
Scrotal US	intra-testicular lesion	115.87
AFP		23.06
bHCG		70.98
LDH		8.30
LFT's		11.23
CBC		10.69
BMP		11.63
CXR		28.52
Rad Ing Orch		479.19
Anesthesia-General		363.72
Peri-operative Services		6,309.00
Pathology Analysis	Malignant-Seminoma	97.38
	(Stage IA, IB, IS, IIA or IIB)	
CT abdomen w/ contrast		257.51
CT pelvis w/ contrast		252.86
CT chest w/ contrast		242.81
Repeat bHCG, AFP, LDH		102.34
**Surveillance**	(Stage IA or IB)	
Level III office visit	q3months x2 years	549.92
	q6 months x2years	274.96
	q year x6 years	412.44
bHCG, AFP, LDH	q3months x2 years	818.72
	q6 months x2years	409.36
	q year x6 years	614.04
CT abdomen and pelvis w/ contrast	q6 months x3years	3,062.22
	qyear x2years	1020.74
CXR	q6 months x3years	171.12
	qyear x2years	57.04
**Chemotherapy**	(Stage IA or IB)	
Carboplatin x1		670.37
plus surveillance costs		
**Radiation**	(Stage IA, IB, IS, IIA or IIB)	
Cost Radiation	10 treatments	3,381.36
plus surveillance costs		
Total Surveillance Group Cost		***15,932.12***
Total Chemotherapy Group Cost		***16,602.49***
Total Radiation Group Cost		***19,313.48***
**Total Average Cost**		**17,282.70**

For Review Only

Scenario F	Finding	Medicare Costs ($)
TSE-felt something		
Level IV new patient		156.47
Scrotal US	intra-testicular lesion	115.87
AFP		23.06
bHCG		70.98
LDH		8.30
LFT's		11.23
CBC		10.69
BMP		11.63
CXR		28.52
Rad Ing Orch		479.19
Anesthesia-General		363.72
Peri-operative Services		6,309.00
Pathology Analysis	Malignant-Non-Seminoma	97.38
	(Stage IA, IB, IS, IIA or IIB)	
CT abdomen w/ contrast		257.51
CT pelvis w/ contrast		252.86
CT chest w/ contrast		242.81
Repeat bHCG, AFP, LDH		102.34
**Surveillance**	(Stage IA or IB)	
Level III office visit	q2 months x2 years	824.88
	q3 months x2 years	549.92
	q6 months x1 year	137.48
	qyear x5 years	343.70
bHCG, AFP, LDH	q2 months x2 years	1,228.08
	q3 months x2 years	818.72
	q6 months x1 year	204.68
	qyear x5 years	511.70
CXR	q2 months x2 years	342.24
	q3 months x2 years	228.16
	q6 months x1 year	57.04
	qyear x5 years	142.60
CT abdomen/pelvis w/ contrast	q4 months x1 year	1,531.11
	q6months x3 years	3,062.22
	qyear x6years	3,062.22
**Chemotherapy**		
BEPx2	(Stage IA or IB)	4,198.75
BEPx3	(Stage IS, IIA or IIB)	6,061.97
or EPx4		

For Review Only

Average chemotherapy cost		5,130.36
plus surveillance costs		
**RPLND**	(Stage IA, IB, IS, IIA or IIB)	
RPLND		982.43
Pathology-RPNLD		215.50
Anesthesia-General		363.72
Anesthesia-Epidural		226.40
Peri-operative Services		6,309.00
Hospital Admission		191.57
Hospital Stay	x3 days	293.43
Hospital Discharge		100.77
plus surveillance costs		
Total Surveillance Group Cost		***21,586.31***
Total Chemotherapy Group Cost		***26,716.67***
Total RPNLD Group Cost		***30,269.13***
**Total Average Cost**		**26,190.70**
**Scenario G**	**Finding**	**Medicare Costs ($)**
No TSE-pain/swelling		
Level IV new patient		156.47
Scrotal US	intra-testicular lesion	115.87
AFP		23.06
bHCG		70.98
LDH		8.30
LFT's		11.23
CBC		10.69
BMP		11.63
CXR		28.52
Rad Ing Orch		479.19
Peri-operative Services		6,309.00
Anesthesia-General		363.72
Pathology Analysis	Seminoma	97.38
	(Stage IIC or III)	
CT abdomen w/ contrast		257.51
CT pelvis w/ contrast		252.86
CT chest w/ contrast		242.81
Repeat bHCG, AFP, LDH		102.34
**Chemotherapy**	Intermediate risk	
BEPx4		8,239.87
AFP		23.06
BhCG		70.98

For Review Only

LDH		8.30
CT chest		242.81
CT abdomen		257.51
CT pelvis	partial response	252.86
PET Scan	positive nodes/mass	1,058.97
Limited Lymphadenectomy		675.49
Pathology Analysis	Malignant	215.50
Anesthesia-General		363.72
Anesthesia-Epidural		226.40
Peri-operative Services		6,309.00
Hospital Admission		191.57
Hospital Stay	x3 days	293.43
Hospital Discharge		100.77
AFP		194.21
BhCG		129.17
LDH		28.08
CT chest		982.43
CT abdomen		675.49
CT pelvis		363.72
**Salvage Chemo**		
VIPx4		9,315.21
**Surveillance**		
Level III office visit	q2 months x1year	412.44
	q3 months x1year	274.96
	q6months x2years	274.96
	qyear x6 years	412.44
CXR	q2 months x1year	171.12
	q3 months x1year	114.08
	q6months x2years	114.08
	qyear x6 years	171.12
bHCG, AFP, LDH	q2 months x1year	614.04
	q3 months x1year	409.36
	q6months x2years	409.36
	qyear x6 years	614.04
CT abdomen/pelvis w/ contrast	q 6 months x2 years	2,041.48
	qyear x8 years	4,082.96
**Total Cost**		**48,876.55**
**Scenario H**	**Finding**	**Medicare Costs ($)**
No TSE-pain/swelling		
Level IV new patient		156.47
Scrotal US	intra-testicular lesion	115.87

For Review Only

AFP		23.06
bHCG		70.98
LDH		8.30
LFT's		11.23
CBC		10.69
BMP		11.63
CXR		28.52
Anesthesia-General		363.72
Rad Ing Orch		479.19
Peri-operative Services		6,309.00
Pathology Analysis	Non-Seminoma	97.38
	(Stage IIC or III)	
CT abdomen w/ contrast		257.51
CT pelvis w/ contrast		252.86
CT chest w/ contrast		242.81
Repeat bHCG, AFP, LDH		102.34
**Chemotherapy**		
BEPx4	Intermediate risk	8,239.87
AFP		23.06
BhCG		70.98
LDH		8.30
CT chest		242.81
CT abdomen		257.51
CT pelvis	partial response	252.86
	resectable lesions	
**RPNLD**		
RPLND		982.43
Pathology-RPNLD	malignancy	215.50
Anesthesia-General		363.72
Anesthesia-Epidural		226.40
Peri-operative Services		6,309.00
Hospital Admission		191.57
Hospital Stay	3 days	293.43
Hospital Discharge		100.77
AFP		226.40
BhCG		194.21
LDH		129.17
CT chest		675.49
CT abdomen		704.57
CT pelvis		982.43

For Review Only
